# Strategies to Improve Glaucoma Compliance Based on Cross-Sectional Response-Based Data in a Tertiary Healthcare Center: The Glauco-Jung Study

**DOI:** 10.5005/jp-journals-10008-1182

**Published:** 2015-09-25

**Authors:** Vishnu S Gupta, Harindersingh Sethi, Mayuresh Naik

**Affiliations:** Consultant, Professor and Head, Department of Ophthalmology, Vardhman Mahavir Medical College and Safdarjung Hospital, New Delhi, India; Consultant and Associate Professor, Department of Ophthalmology, Vardhman Mahavir Medical College and Safdarjung Hospital, New Delhi, India; Postgraduate Resident (3rd Year), Department of Ophthalmology, Vardhman Mahavir Medical College and Safdarjung Hospital, New Delhi, India

**Keywords:** Compliance, Cost of medications, Glaucoma.

## Abstract

**Purpose:** To elucidate compliance rates among glaucoma patients in a tertiary healthcare center, reasons for noncompliance and response-based-solutions to improve compl iance in the same cohort.

**Materials and methods:** In the Glauco-Jung study, a cross-sectional descriptive epidemiological one, information was obtained from 500 patients from 1st January, 2014 to 30th June, 2014. Patients were intercepted at entry point where they get their intraocular pressure (IOP) checked, wherein they were asked to fill an exhaustive questionnaire. At the same setting, they were also asked to demonstrate how they (or their relatives or helpers) instill eyedrops, following which any irregularities were brought to notice and corrected. Finally, they were also asked any suggestions to improve compliance to medications.

Noncompliance rates were determined based on the number of patients who did not instill anti-glaucoma medications as per prescribed dosage or frequency schedule. Noncompliance rates were then evaluated by the Chi-square test for any association with distributions based on various parameters.

**Results:** In case of a positive association, correlation coefficient was further calculated to know the strength of this association. No association was observed in distributions based on diet, associated co-morbidities, daily dosage frequency and side-effects experienced by patients. Positive association was noted in distributions based on age, sex, duration of treatment, social structure and number of medications (p < 0.05); but correlation coefficients were very weak (c < 0.3). Cost of medications not only had positive association but also had a very strong correlation coefficient (c = 0.9188), proving that cost of medications had a modest bearing on compliance rates.

**Conclusion:** The Glauco-Jung study concluded that besides availability of medications at reasonable cost, simplification of treatment regimen and interactive health education appear to be the most important factors for improving compliance so that patients do not feel guilty or inadequate because they have problems while administering their eyedrops.

**How to cite this article:** Gupta VS, Sethi H, Naik M. Strategies to Improve Glaucoma Compliance Based on Cross-Sectional Response-Based Data in a Tertiary Healthcare Center: The Glauco-Jung Study. J Curr Glaucoma Pract 2015;9(2):38-46.

## INTRODUCTION

Noncompliance with medical therapy has long been recognized as an important limiting factor in the medical management of many chronic diseases.^1,2^ Patients with glaucoma who have lower rates of compliance are presumed to be at greater risk of developing progressive visual loss.^3^ Rates of compliance with therapeutic regimens for chronic diseases, including glaucoma, may be as low as 50%; and noncompliance has been associated with an increase in hospital admissions, length of stay, and healthcare costs.^4^

The present Glauco-Jung study is an effort to elucidate the compliance rates among glaucoma patients and the reasons for noncompliance as well as goes a step further in providing possible solutions to improve compliance in the same cohort.

## MATERIALS AND METHODS

Information was obtained from 500 patients attending the speciality glaucoma clinic from 1st January, 2014 to 30th June, 2014. The patients were intercepted at the entry point where they get their intraocular pressure (IOP) checked, wherein they were asked to fill a questionnaire. The questions that were used explored the patients’ knowledge of their condition and its treatment, whether their compliance was good or bad and the reasons for this, how they administered their drops, any problems encountered, and whether they would welcome a compliance aid if a suitable one was available. At the same setting, they were also asked to demonstrate how they (or their relatives or helpers) instill eyedrops, following which any irregularities were brought to notice and corrected. Finally, they were also asked any suggestions to improve compliance to medications.

Noncompliance rates were determined based on the number of patients who did not instill anti-glaucoma medications as per prescribed dosage or frequency schedule. Noncompliance rates were then evaluated by the Chi-square test for any association with distributions based on various parameters.

## RESULTS

**Graph 1:**
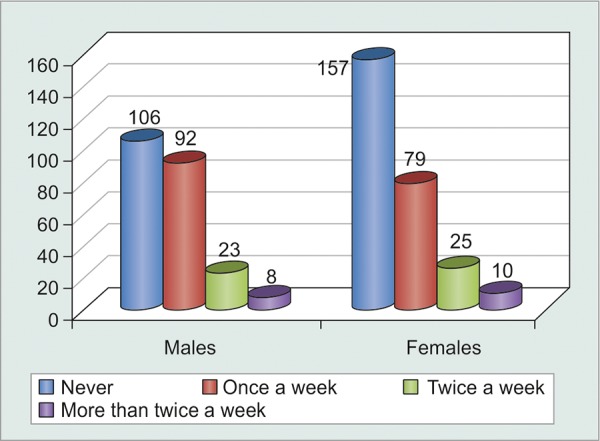
Graphical sex-wise distribution of noncompliant patients

Even though females reporting to the glaucoma clinic outnumbered males, the difference was not statistically significant (p = 0.15). There was a statistically significant difference (p = 0.053) in sex distribution of compliance, wherein a greater proportion of males were non-compliant as compared to females. However, the correlation, as such, was very weak (correlation coefficient = 0.123) (Table 1 and Graph 1).

**Graph 2:**
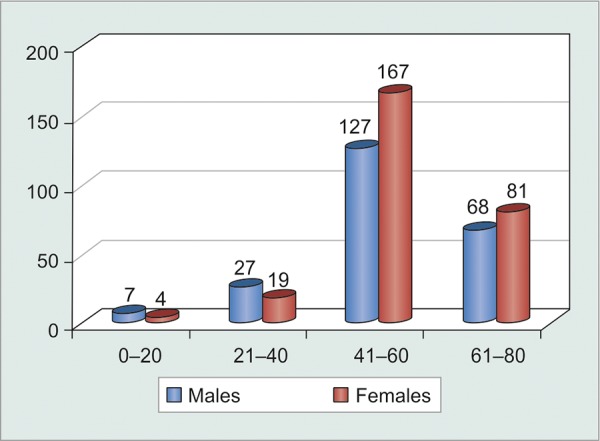
Graphical age-wise distribution of patients

The major chunk was, however, formed by the age group 41 to 60 years, i.e. the so called ‘economically-productive-age-group’. This was the age group where our machinery to increase compliance, should be targeted (Table 2 and Graph 2).

**Graph 3:**
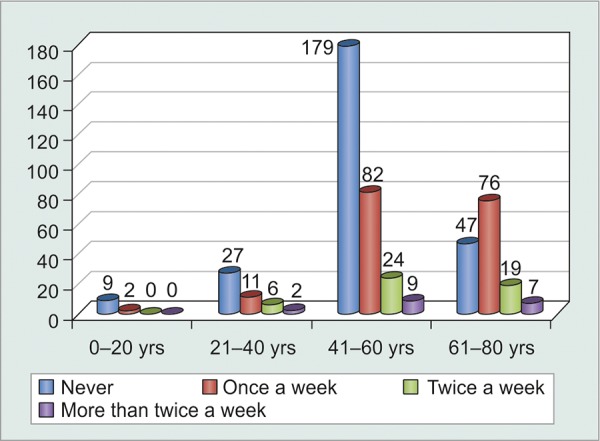
Graphical age-wise distribution of noncompliant patients

Age was a statistically significant factor affecting compliance (p < 0.0001). However, there was only a weak correlation between age and noncompliance (c = 0.277), indicating that increasing age did not affect compliance adversely (Table 3 and Graph 3).

**Graph 4:**
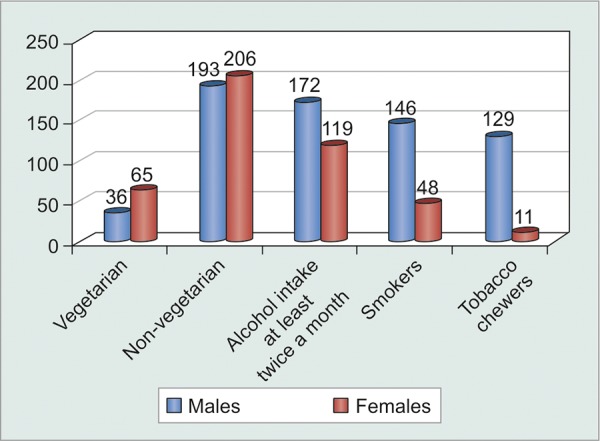
Graphical diet-wise distribution of patients

**Graph 5:**
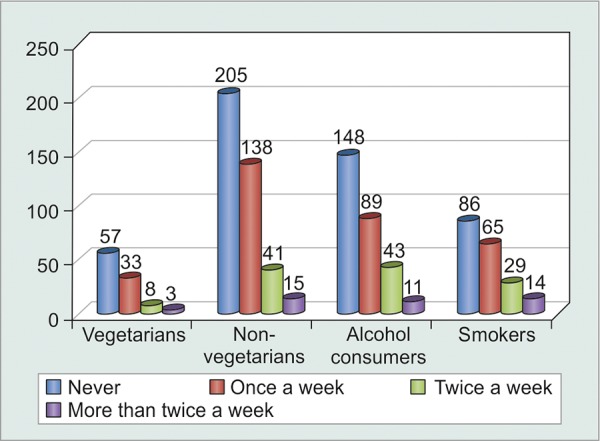
Graphical distribution of noncompliant patients

Urban population in Delhi shows a preference of non-vegetarian diet while more than half of those presenting to the glaucoma clinic agreed to have had alcohol intake (60-180 ml) more than twice a month. However, compliance rates were not significantly affected (p = 0.1551) by diet, alcohol and tobacco consumption (Table 4 and Graphs 4 and 5).

**Table 1:** **Table 1:** Tabulated sex-wise distribution of noncompliant patients

*Patients* *forgetting* * to instill* * eyedrops*		*Never*		*Once* * a week*		*Twice* * a week*		*More than* *twice a* *week*		*Total*	
Males		106		92		23		8		229	
Females		157		79		26		10		271	
Total		263		171		49		18		500	

**Table 2:** **Table 2:** Tabulated age-wise distribution of patients

*Age (years)*		*Males*		*Females*		*Total*	
0-20		7		4		11	
21-40		27		19		46	
41-60		127		167		294	
61-80		68		81		149	
Total		229		271		500	

**Table 3:** **Table 3:** Age-wise distribution of noncompliant patients

*Patients* *forgetting to* * instill eyedrops*		*Never*		*Once* * a week*		*Twice* * a week*		*More than* *twice a* *week*		*Total*	
0-20 years		9		2		10		0		11	
21-40 years		27		11		6		2		6	
41-60 years		179		82		24		9		294	
61-80 years		47		76		19		7		149	
Total		262		171		49		18		500	

**Table 4:** **Table 4:** Tabulated diet-wise distribution of noncompliant patients

*Patients* *forgetting to* * instill* * eyedrops*		*Never*		*Once* * a week*		*Twice* * a week*		*More* *than* * twice a* * week*		*Total*	
Vegetarians		57		33		8		3		101	
Non-vegetarians		205		138		41		15		399	
Alcohol consumers		148		89		43		11		291	
Smokers		86		65		29		14		194	

**Graph 6:**
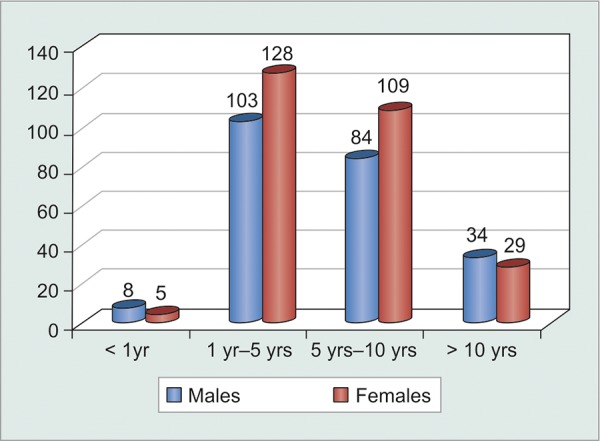
Graphical distribution of patients based on duration under treatment

**Graph 7:**
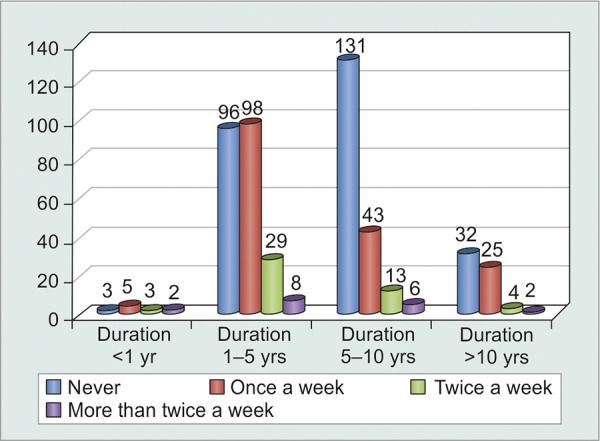
Graphical distribution of noncompliant patients based on duration under treatment

The greatest number of patients following up regularly for glaucoma clinic evaluation were those who were diagnosed recently, i.e. within 1 to 5 years. However, duration of treatment apparently did not have any significant bearing on patient compliance (p = 0.3) (Table 5 and Graph 6). Statistical analysis of noncompliant patients against their duration under treatment showed that patients were highly compliant initially during their course of treatment, while they tended to become non-compliant (p < 0.0001) as they became chronic visitors of the glaucoma clinic. However, correlation coefficient was quite weak (c = 0.276), thus, proving that it could not be generalized that chronic glaucoma patients became noncompliant over time (Table 6 and Graph 7).

**Graph 8:**
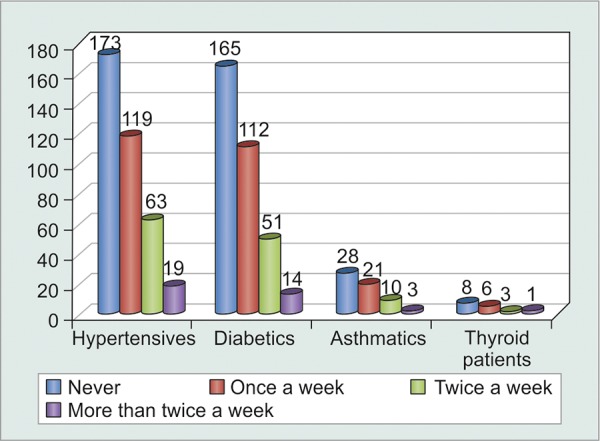
Graphical distribution of noncompliant patients with associated co-morbidities

All patients screened for any associated medical disorders were then referred to respective medical specialty clinics and urged to follow-up and comply with their medications. Patients compliant with their medications for medical disorders generally ended up being compliant for their medications for ophthalmic disorders as well. Unfortunately, this could not be statistically proved (p = 0.9) (Tables 7 and 8, Graph 8).

**Graph 9:**
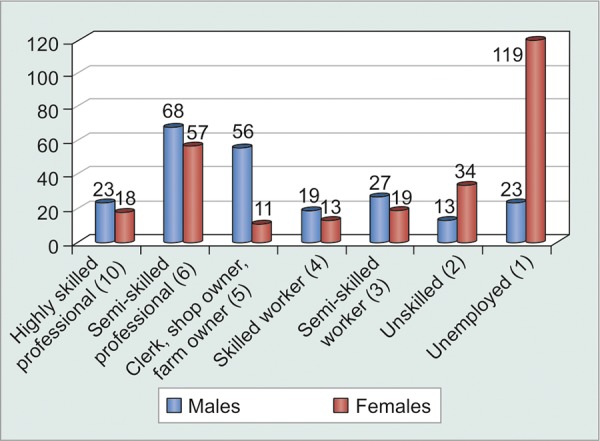
Graphical distribution of patients based on Kuppuswamy classification of social structure in India

**Graph 10:**
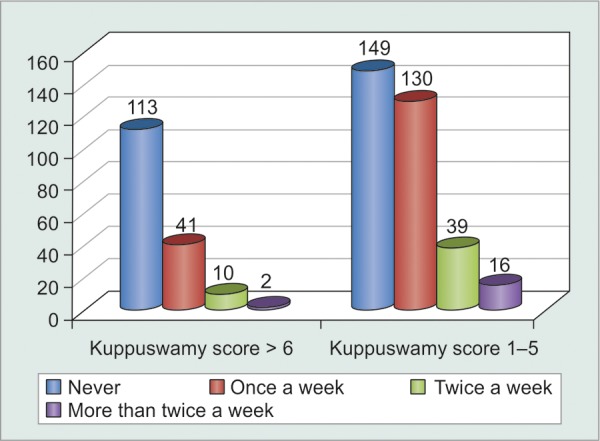
Graphical distribution of noncompliant patients based on Kuppuswamy classification for social structure in India

**Fig. 1:**
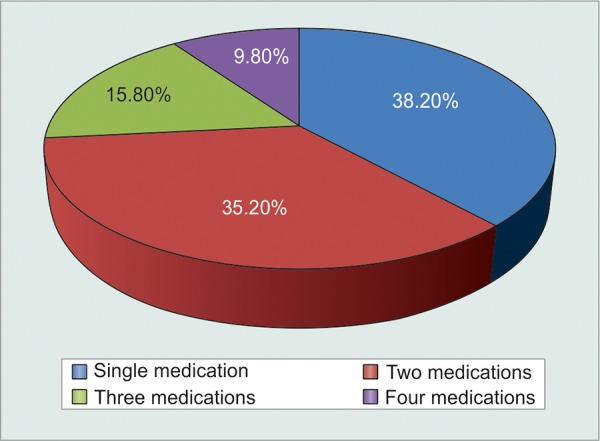
Graphical distribution of patients based on number of medications

**Graph 11:**
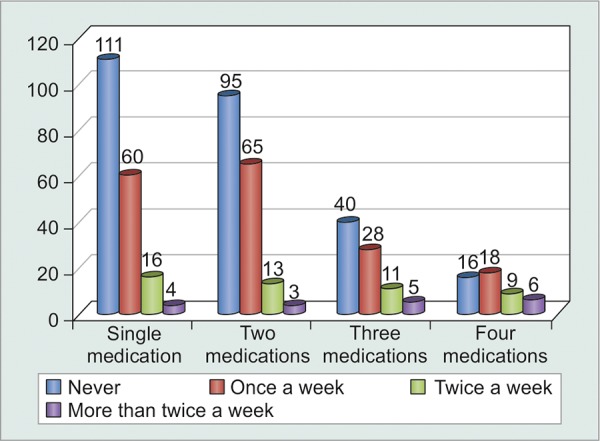
Graphical distribution of noncompliant patients based on number of medications

Since Indian social structure is adverse to females, illiteracy and unemployment were far more abundant in females as compared to males (Table 9 and Graph 9). Statistical analysis proved that illiterate and unemployed patients were less motivated to comply (p < 0.001) with treatment as compared to their literate and employed counterparts. However, correlation was still weak enough (c = 0.221) to suggest that literacy could definitely ensure patient compliance (Table 10 and Graph 10). Greater the number of medications that patients were started on, greater was the tendency to forget instilling some of them, and hence greater was their noncompliance (p = 0.0016). However, weak correlation (c = 0.225) suggested that there were a good number of motivated patients who were on multiple medications and were yet compliant enough (Fig. 1, Table 11 and Graph 11).

**Table 5:** **Table 5:** Tabulated distribution of patients based on duration under treatment

		*No. of patients*					
*Duration*		*Males*		*Females*		*Total*	
< 1 year		8		5		13	
1-5 years		103		128		231	
5-10 years		84		109		193	
>10 years		34		29		63	
Total		229		271		500	

**Table 6:** **Table 6:** Tabulated distribution of noncompliant patients based on duration under treatment

*Patients forgetting* *to instill eyedrops*		*Never*		*Once* * a* * week*		*Twice* * a* * week*		*More* * than* *twice a* *week*		*Total*	
Duration < 1 year		3		5		3		2		13	
Duration 1-5 years		96		98		29		8		231	
Duration 5-10 years		131		43		13		6		193	
Duration > 10 years		32		25		4		2		63	
Total		262		171		49		18		500	

**Table 7:** **Table 7:** Tabulated distribution of patients with associated co-morbidities

*Associated co-morbidity*		*No. of patients*	
Hypertension		374	
Diabetes		342	
Asthma		62	
Thyroid disorders		18	

**Table 8:** **Table 8:** Tabulated distribution of noncompliant patients with associated co-morbidities

*Patients forgetting to* *instill eyedrops*		*Never*		*Once a week*		*Twice a week*		*More than* *twice a week*		*Total*	
Hypertensives		173		119		63		19		374	
Diabetics		165		112		51		14		342	
Asthmatics		28		21		10		3		62	
Thyroid patients		8		6		3		1		18	

**Table 9:** **Table 9:** Tabulated distribution of patients based on Kuppuswamy classification for social structure in India

*Kuppuswamy classification*					
		*Males*		*Females*	
Highly skilled professional^10^		23		18	
Semi-skilled professional^6^		68		57	
Clerk, shop owner, farm owner^5^		56		11	
Skilled worker^4^		19		13	
Semi-skilled worker^3^		27		19	
Unskilled^2^		13		34	
Unemployed^1^		23		119	
Total		229		271	

**Fig. 2:**
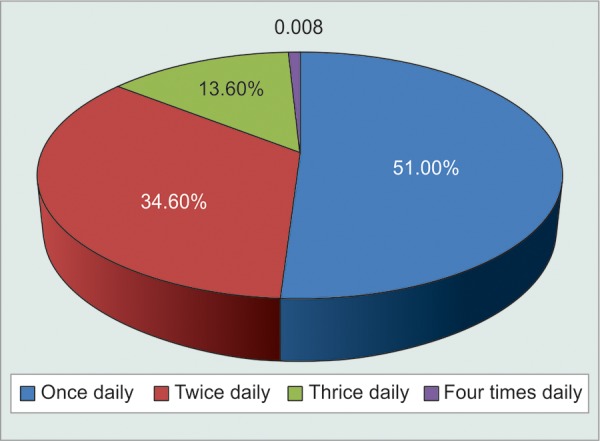
Graphical distribution of patients based on dosage frequency

**Graph 12:**
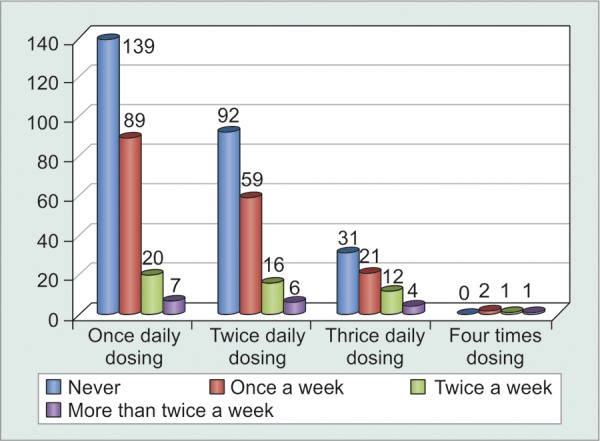
Graphical distribution of noncompliant patients based on dosage frequency

Even though patients themselves claimed that it was difficult to adhere to multiple dosage timings each day and that they found it easier to comply to single dose frequency, the results could not be proven to be statistically significant (p = 0.06) (Fig. 2, Table 12, Graph 12). Analysis of relationship between monthly cost of medications and irregularities in buying medications proved that their correlation was statistically significant (p = 0.0274). A highly positive correlation coefficient of 0.9188 suggested that as monthly cost of medications increased, patients became more irregular in buying them. In fact, this was the most important observation in the study. Hence, there is an urgent need to provide as many of the most effective anti-glaucoma medications at government dispensaries at highly subsidized rates (Table 13).

**Graph 13:**
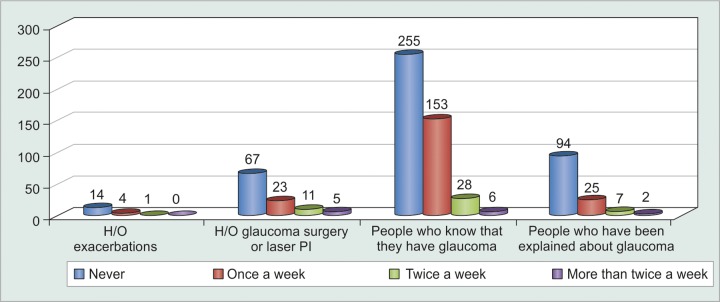
Graphical distribution of noncompliant patients based on previous history

Patients who had had exacerbations of acute or chronic glaucoma, or who had undergone either laser peripheral iridectomy or glaucoma filtering surgery were more prone to remain compliant on their medications as compared to others. Also, only 51% patients knew they had glaucoma, i.e. 49% were unaware of this fact despite being on regular follow-up to the glaucoma clinic. In fact, 34% patients knew that they had glaucoma but had no clue of what glaucoma was or its adverse visual consequences. Hence, compliance was significantly increased (p = 0.0051) among patients after they were explained about glaucoma and the need for medications as well as their compliance to it (Tables 14 and 15, Graph 13). The most common side-effects experienced were redness, burning and itching. However, a meager number of patients were found to discontinue their medications without seeking medical advice. Rest patients were comfortably relieved off their discomfort with lubricants and decongestants (Tables 16 and 17).

**Table 10:** **Table 10:** Tabulated distribution of noncompliant patients based on Kuppuswamy classification for social structure in India

*Patients* * forgetting* *to* *instill* * eyedrops*		*Never*		*Once* * aweek*		*Twice a* * week*		*More* *than* *twice* *a* *week*		*Total*	
Kuppuswamy score > 6		113		41		10		2		166	
Kuppuswamy score 1-5		149		130		39		16		334	
Total		262		171		49		18		500	

**Table 11:** **Table 11:** Tabulated distribution of noncompliant patients based on number of medications

*Patients* *forgetting to* *instill eyedrops*		*Never*		*Once a* *week*		*Twice a* *week*		*More* *than* *twice a* * week*		*Total*	
Single medication		111		60		16		4		191	
Two medications		95		65		13		3		176	
Three medications		40		28		11		5		84	
Four medications		16		18		9		6		49	
Total		262		171		49		18		500	

### Miscellaneous Results (Figs 3 to 5)

Based on the questionnaire, the Glauco-Jung study reiterated nine most important reasons for noncompliance of eyedrops, in descending order of importance and frequency (correlated to number of patients stating it a their reason for noncompliance):

**Table 12:** **Table 12:** Tabulated distribution of noncompliant patients based on dosage frequency

*Patients* *forgetting to* * instill eyedrops*		*Never*		*Once* * a* * week*		*Twice* * a week*		*More than* *twice a* *week*		*Total*	
Once daily dosing		139		89		20		7		255	
Twice daily dosing		92		59		16		6		173	
Thrice daily dosing		31		21		12		4		68	
Four times dosing		0		2		1		1		4	
Total		262		171		49		18		500	

- To difficult to follow the complex regimen.
- Unpleasant outcomes or side-effects.
- Too expensive.
- Lack of trust that the eyedrops really work.
- Blinking too much: Discussion with patients indicated that lack of confidence was a major factor, particularly fear of prodding the eye. As a result, the bottle was often held too far from the eye, making the aim more difficult and encouraging the blink reflex.
- Cannot remember whether drops were put.
- Too hard to squeeze the bottle just right, especially after refrigeration.
- Too hard to stay on target either because of poor visibility of dropper tip or shaky hands.
- No assistance for old aged people to put the eyedrops.

**Table 13:** **Table 13:** Tabulated distribution of noncompliant patients based on cost of medications

*Cost of medications* *per month*		*No. of patients*		*No. of patients* *irregular in buying* *eyedrops*	
< Rs.100		0		0	
Rs101-500		282		31	
Rs 501-1000		177		29	
Rs1001-1500		32		7	
> Rs1501		9		5	
Total		500		72	

**Table 14:** **Table 14:** Tabulated distribution of patients based on previous history

		*No. of patients*	
Ever had oral medications		68	
Ever had exacerbations		19	
CGHS Beneficiaries		368	
(Central Govt Health Scheme)			
Undergone glaucoma surgery		28	
Undergone laser PI		78	
Undergone any other eye surgery		278	
Patients who know that they have glaucoma		442	
Patients who have been explained		128	
about glaucoma			
Patients who have had their doubts cleared		78	

**Table 15:** **Table 15:** Tabulated distribution of noncompliant patients based on previous history

*Patients* *forgetting to* *instill eyedrops*		*Never*		*Once a* *week*		*Twice a* *week*		*More* *than* *twice a* *week*		*Total*	
H/o exacerbations		14		4		1		0		19	
H/o glaucoma surgery or PI		67		23		11		5		106	
Patients who know that they have glaucoma		255		153		28		6		442	
Patients who have been explained about glaucoma		94		25		7		2		128	

**Table 16:** **Table 16:** Tabulated distribution of patients based on the side-effects experienced

*Side-effects experienced*		*No. of patients*	
Redness		348	
Burning		382	
Itching		328	
Pain in eyes		68	
Palpitations		12	
Breathing difficulty		23	

**Table 17:** **Table 17:** Tabulated distribution of noncompliant patients based on side-effects experienced

*Side-effects* *experienced*		*No. of patients*		*No. of patients* *stopping medication* *without medical* *advice*	
Redness		348		21	
Burning		382		27	
Itching		328		9	

**Fig. 3:**
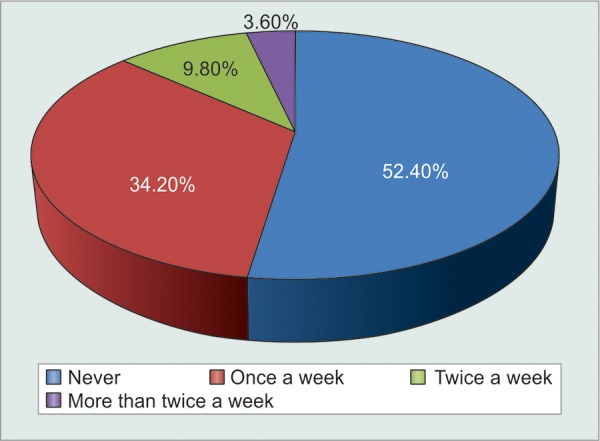
Graphical distribution of noncompliant patients who miss instilling eyedrops

**Fig. 4:**
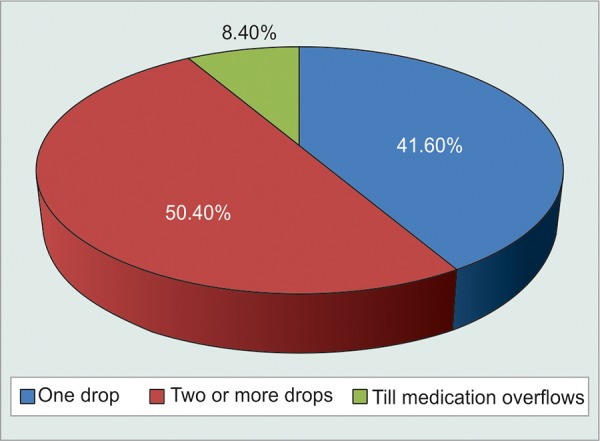
Graphical distribution of number of eyedrops instilled by patients

There also exists a certain minimal amount of KAP-GAP among glaucoma patients.^5^ The ‘KAP-GAP’ refers to the gap between knowledge, attitudes and practice; and is not specific to any age group, gender, community, region or population. It is both real and resilient, and has long been concerned about a persistent gap which exists when knowledge and favorable attitude do not lead toward adoption of a practice. In spite of the fact that the patients knew that they had glaucoma, and that discontinuation or irregularity of eyedrops would lead to progressive visual loss, there were 1.8% (8/500) patients who were noncompliant for untenable reasons.

**Fig. 5:**
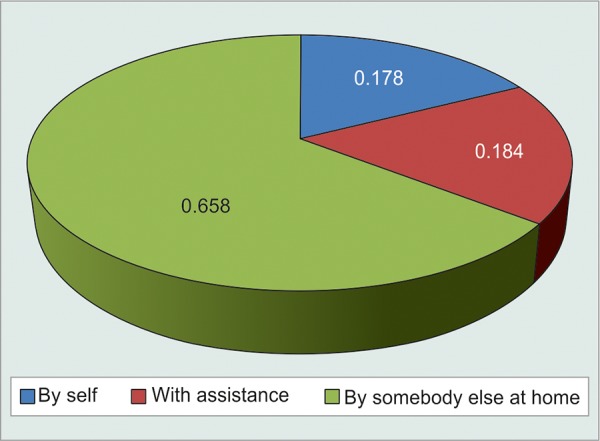
Graphical distribution of patients based on assistance while instilling eyedrops

## DISCUSSION

Most meta-analyses yielding cost-benefit ratios, that harness measures of medical usage, have been tampered by the lack of certain consistency in standards relating to definitions and quantifications used to describe the ‘concepts of drug compliance’.^6^ Health outcomes analysts need practical and operationally useful definitions that would help in standardizing the available results, in quantifying a common benchmark for comparing and amalgamating results, and for assisting the development of potent and efficient intervention strategies to upgrade patient drug compliance. The medication compliance and persistence work group of the international society for pharmacoeconomics and outcomes research (ISPOR) formulated definitions for compliance and persistence during 3 years of international review and discussion. Compliance is, thereby defined as ‘the extent to which a patient acts in accordance with the prescribed interval and dose of a dosing regimen’, and thus refers to the act of conforming to the recommendations made by the provider with respect to timing, dosage and frequency of medication taking. Persistence may be defined as ‘the duration of time from initiation to discontinuation of therapy’.^7^

Untreated glaucoma ensures progressive damage to the optic nerve, along with progressive visual field defects even including loss of the final surviving ‘island of vision’ and eventually, blindness.^8^ Even then, the single most influential deterrent to the successful arrest of glaucoma progression is poor patient compliance with the treatment regimen.^9^ Since glaucoma produces few symptoms, there is meager motivation for patients to be consistent with treatment; especially when in the initial stages, when vision-threatening complications have not begun, the only symptoms experienced would be the side-effects of the medication.^10^ The aim of therapy for glaucoma is to preserve vision with minimal side-effects and inconvenience.^11^

A major decisive factor of compliance with anti-glaucoma treatment is patient’s knowledge of having glaucoma and the insight regarding the potential for blindness: the more grave their visual loss, the more probable it is that patients will comply with drug schedule as medicated.^10^ Compliance is further restrained by the physical ineptitude of patients to use eyedrops adequately, with difficulty expelling the drop from the vial, instilling the drop correctly, and blinking while insertion.^12^ Such technical difficulties are referred to as ‘discompliance’. As patients were hesitant to bring up these hindrances with healthcare personnel, the medical fraternity involved with researching ways of bettering patient compliance, were pretty uninformed of the hindrances associated with as meager a task as eye drop instillation. Discompliance is even less realized and admitted than noncompliance. Perceptions about patient compliance, and hence remedies for noncompliance including alternative drug combinations or dosage schedules, are often based on clinical judgments and measurements of IOP, weight of drug vial when the patient reports for follow-up, investigative reports as well as the ophthalmologist’s subjective opinion of the patient.^3^ However, noncompliant patients cannot be identified precisely based on these grounds. In addition, there exists only a fairly limited correlation between the ophthalmologists presumptions about patient’s compliance and the results of monitoring. Since, these measures are not capable of accurately indicating patient compliance, an eyedrop monitor is required for identification of patients who default from therapy. Using an unobtrusive eyedrop monitor as an objective measure, Kass et al found considerable over-reporting of compliance with glaucoma medication in an interview situation.^13^

### The Glauco-Jung study: Strategies to Improve Compliance

Clinical outcomes of treatment are affected not only by how much patients adhere to their prescriptions but also by how long they take their drugs as per dosage schedule. Thus, compliance and persistence should be defined and quantified separately to characterize medication-taking behavior comprehensively. Progression of the disease often results in addition of more efficacious or more potent drugs to the already existing drug combination, which may be inappropriate and unnecessary if the progression is due to noncompliance rather than to treatment failure.^14,15^ In addition, necessary glaucoma surgery may be reviewed if noncompliant patients have an IOP at clinic visits that is fairly low as compared to their degree of noncompliance resulting from instilling the medication only during the preceding hours.^14,15^

- *Patient education:* The usefulness of a 6-minute videotape in educating 98 patients with glaucoma about their disease as evaluated by Rosenthal et al showed that patients knowledge was not only significantly improved immediately after viewing (p < 0.001) but even after 6 months, patients with glaucoma retained more knowledge of the disease than the controls.^16^
- *Regimen improvisation:* Streamlining a regimen to the patient’s lifestyle.
- *Fixed drug combinations:* Interfere significantly less with daily life in terms of activity limitations and side-effects.
- *Decreasing frequency of dosage:* Patients report significantly fewer missed doses while using once daily preparations as against twice daily preparations of the same drug in another formulation, suggesting that, when appropriate, patients appreciate having their therapy simplified.
- *Compliance aids:* Xal-Ease dispenser from Pfizer for latanoprost products . ‘Eyot’ dispensers available from Alcon for travoprost products. Opticare and Opticare arthro dispensers are designed for people whose hands shake, or have difficulty placing or squeezing the bottle. The Unidoser (Mystic pharmaceuticals) uses a patient-replaceable cartridge system to deliver metered unit doses of ophthalmic medication to the ocular surface. Allergan’s Lumigan compliance aid features a light timed to flash and an optional audible alarm.
- *Tending to possible side-effects:* An emergent advance in therapy is a device that can turn medication into a mist rather than a drop, which is being developed by Optimyst systems (West Islip, New York). This may circumvent the squeamishness some patients have about taking eyedrops.

## CONCLUSION

The Glauco-Jung study concluded that no doubt the barrier to free communication can be broken down if ophthalmologists take a lead. There is probably also a need for an ‘awareness campaign’ aimed at patients, so that they do not feel guilty or inadequate because they have problems while administering their eyedrops. Simplification of the treatment regimen and interactive health education appear to be the most important factors for improving compliance. The availability of a suitable device will also probably help patients achieve safe administration and improve compliance.
